# Signal Analysis on Strings for Immune-Type Pattern Recognition

**DOI:** 10.1002/cfg.367

**Published:** 2004-02

**Authors:** Nikolaos D. Atreas, Costas Karanikas, Persefoni Polychronidou

**Affiliations:** Department of Informatics Aristotle University of Thessaloniki Thessaloniki 54124 Greece

## Abstract

We use wavelet-type discrete transforms for signal analysis on strings of finite length. We apply these transforms for edge and hidden Markov process detection. We also present new approaches for string matching and for measures of the diversity of chaotic strings.


**Comparative and Functional Genomics**
*Comp Funct Genom* 2004; **5**: 69–74.
Published online in Wiley InterScience (www.interscience.wiley.com). **DOI:** 10.1002/cfg.367

**Conference Review**

### Signal analysis on strings for immune-type pattern recognition

Nikolaos D. Atreas, Costas Karanikas* and Persefoni Polychronidou
*Department of Informatics, Aristotle University of Thessaloniki, Thessaloniki 54124, Greece*

*Correspondence to: Costas Karanikas, Department of Informatics, Aristotle University of Thessaloniki, Thessaloniki 54124, Greece. E-mail: karanika@csd.auth.gr*

Received: 10 November 2003
Revised: 27 November 2003
Accepted: 28 November 2003

### Abstract

**We use wavelet-type discrete transforms for signal analysis on strings of finite length. We apply these transforms for edge and hidden Markov process detection. We also present new approaches for string matching and for measures of the diversity of chaotic strings. Copyright © 2004 John Wiley & Sons, Ltd.**

**Keywords: immune-inspired computational systems; discrete tree transform; edge detection; hidden Markov model; string matching**

### Introduction

The immune system is one of the most effective pattern recognition systems. This system deals with RNA strings on proteins and viruses and is involved in several operations and recognition modes capable of:

- Detecting local particularities.
- Detecting diversity.
- Applying threshold operations.
- Making edge detection.
- Discriminating and taking decisions.

In order to partially model these recognition capabilities, we introduce new discrete transforms, providing an effective background for immune-type computational applications on spaces of strings. All these transforms exploit local information, as does the immune system.

In this review, we discuss how the discrete tree transform (DTT), introduced in the Karanikas and Proios [10] model of antigen processing, and then we show (as in Atreas *et al.* [2]) how DTT achieves edge detection. We explain how we apply DTT structures to strings to detect hidden Markov processes, we introduce a measure for the diversity of strings based on fractal dimension formulae, as in Bisbas and Karanikas 1990 [6], and we present a novel string matching method based on analytic number theory.

As Felix Browder, the President of the American Mathematical Society, said in his Retiring Presidential Address [4]:

> In molecular biology, mathematics has a much greater role to play than people realize, even though mathematics has had, for example, a significant effect on the course of the genome project. There will be an even larger effect when it comes to analysing how the genome actually creates living cells ... The rituals of classical statistics no longer suffice to deal with many problems that people face, especially when they have large masses of data — and large masses of data are the basic ingredient of the modern world.

In this short review we hope to make clear that new mathematical methods applied on strings of biological data could provide a new era for bioinformatics.

### Antigen processing and the discrete tree transform (DTT)

Antigen processing is an important operation used by the immune system to provide total success of recognition and destruction of any non-self intruder. The operation can be considered as cutting

Copyright © 2004 John Wiley & Sons, Ltd.

the antigen into pieces called antigenic peptides (a peptide is a small protein). Peptides usually represent local singularities of the antigen.

In this section we introduce the DTT [10] and examine its properties.

### Definition 1

Let $p = 2, 3, \ldots$, the *p-adic approximation* of a non-negative data collection $T = \{t_1, \ldots, t_{p^N}\}$ is given by:

$$\left\{ R_{n,k}(T) = \sum_{r=(k-1)p^{N-n}+1}^{kp^{N-n}} t_r, \quad k = 1, \ldots, p^n, \quad n = 0, \ldots, N \right\}.$$

Obviously, the collection:

$$\{R_{n,k}(T), k = 1, \ldots, p^n, n = 0, \ldots, N\}$$

has a $p$-adic tree structure with $N + 1$ generations, such that: $R_{0,1}(T)$ corresponds to the initial node of the tree; each $R_{n,k}(T), n = 1, \ldots, N - 1$ corresponds to the $k$ node of the $n$th generation and $R_{N,k}(T)$ is the $k$ branch (or leaf) of the last generation.

### Definition 2

The *walks* $a_{n,k}(T)$ are the following real numbers:

$$a_{n,k}(T) = \begin{cases} 0, & R_{n-1,\left[\frac{k}{p}\right]}(T) = 0 \\ \dfrac{R_{n,k}(T)}{R_{n-1,\left[\frac{k}{p}\right]}(T)}, & R_{n-1,\left[\frac{k}{p}\right]}(T) \neq 0 \end{cases}$$

$$n = 1, \ldots, N, k = 1, \ldots, p^{\mathrm{n}},$$

where for any real number $x$, $[x]$ is the smallest integer less than or equal to $x$.

The DTT of $T$ is the collection of all walks, $a_{n,k}(T)$ as above.

Obviously, DTT cuts data into successively smaller and smaller p-adic pieces (peptides), mimicking antigen processing. Local singularities are represented by sets of ratios called walks. Walks, as do peptides, represent local singularities and allow the reconstruction of the initial data. Indeed:

### Proposition 1

The DTT of $T = \{t_1, \ldots, t_{p^N}\}$ satisfies the multiplication formula:

$$R_{n,k}(T) = a_{n,k}(T)a_{n-1,[k/p]}(T) \ldots a_{1,[k/p^{n-1}]}(T)R_{0,1}(T),$$
$$n = 1, \ldots, N, k = 1, \ldots, p^n.$$

Note that $R_{N,k}(T) = t_k$. Thus, for $n = N$, the formula reconstructs the initial data set (leaves of the tree), while for $n < N$ it reconstructs the branches of the tree. The notion of DTT can be easily extended on finite strings, as shown in the following:

#### Example

The binary walks of the data $\{c, t, g, c, a, a, a, t\}$ are the following:

$$\left\{ \left\{ \frac{2c+g+t}{3a+2c+g+2t}, \frac{3a+t}{3a+2c+g+2t} \right\}, \left\{ \frac{c+t}{2c+g+t}, \frac{c+g}{2c+g+t}, \frac{2a}{2c+g+t}, \frac{a+t}{2c+g+t} \right\}, \left\{ \frac{c}{c+t}, \frac{t}{c+t}, \frac{g}{c+g}, \frac{c}{c+g}, \frac{1}{2}, \frac{1}{2}, \frac{a}{a+t}, \frac{t}{a+t} \right\} \right\}.$$

As do antigenic peptides, the walks show the singularities of the processed antigen and can reconstruct it, e.g. to reconstruct the first element of $\{c, t, g, c, a, a, a, t\}$, multiply the related walks:

$$(3a + 2c + g + 2t)\frac{2c + g + t}{3a + 2c + g + 2t} \times \frac{c + t}{2c + g + t}\frac{c}{c + t} = c.$$

DTT has several interesting properties, which we shall see next.

### How DTT achieves edge detection

### Edge detection on 2D-plane curves

Edge detection of time series is a computational process consisting of operations aiming to detect extreme changes in the shape of a pattern. Since operations of DTT are capable of erasing short local variabilities and capturing the relevant

Copyright © 2004 John Wiley & Sons, Ltd. 

extreme points, we have presented a method for edge detection of time series [2]. In this section we use DTT for detecting the singularities of 2D-plane curves: $T = \{(x_1, y_1), \ldots, (x_{p^N}, y_{p^N})\}$, where $p$ is a prime number and $N = 2, 3, \ldots$

### Definition 3

The *p-adic approximation* of $T$ is given by the complex numbers:

$$\left\{R_{n,k}(T) = \sum_{r=(k-1)p^{N-n}+1}^{kp^{N-n}} x_r + i \sum_{r=(k-1)p^{N-n}+1}^{kp^{N-n}} y_r, \quad k = 1, \ldots, p^n, n = 0, \ldots, N\right\}.$$

For any $n = 1, \ldots, N-1$, the norm of the $n$th $p$-adic approximation of $T$ is given by the formula:

$$\|V_n(T)\| = \frac{1}{(2^N - 1)2^{N-n}} \sum_{k=1}^{2^n-1} \|\omega_{n,k}(T)\|_2$$

where $\omega_{n,k}(T) = \dfrac{R_{n,k}(T) - R_{n,k+1}(T)}{2^{N-n}}$ and $\|.\|_2$ is the usual Euclidean norm. We shall denote by $||V(T)||$ the norm $||V_N(T)||$.

### Proposition 2

(a) There exists a unique index $1 < n_0 < N$, such that $|\|V_{n_0}(T)\| - \|V(T)\||$ is minimum.
(b) Let $n_0$ be the index of T as in (a); if $P_{n_0,k}$ are the points in plane represented by the complex numbers $R_{n_0,k}(T)$, then the set:

$$J_k(T) = \left\{k : sign\left(\frac{\langle P_{n_0,k}P_{n_0,k+1}, P_{n_0,k+1}P_{n_0,k+2}\rangle}{\|P_{n_0,k}P_{n_0,k+1}\|_2 \|P_{n_0,k+1}P_{n_0,k+2}\|_2}\right) = -1, k = 1, \ldots, 2^{n_0-1} - 2\right\}$$

determines the position of the relevant extreme points of the $n_0$-approximation of $T$, where $\langle , \rangle$ is the usual scalar product.
(c) The set $\{t_{\alpha(s)} : a(s) = p^{N-n}s, s \in J_k(T)\}$ determines the locations of the main edges of the graph $T$.

**Proof**

See [2].

**Example**

We randomly select a curve of the plane consisting of 121 points (Figure 1). Applying Proposition 2, we get the extreme edges of the curve (Figure 2).

### Edge detection of 2D-images

The simplest way to model the binding energy between proteins is in terms of a bilinear form (mechanical/chemical energy form) (see [12,13]). The energy bilinear form is determined by a real rectangular matrix $M$. Using the *SVD* analysis of the matrix $M$:

$$M \cong sLR^T, L^T L = I, R^T R = I$$

where the *singular vectors* $L$, $R$ can be considered as a mathematical model of 'antibody probes', while the real number $(-s)$ is their binding energy, two-dimensional images are reduced to two one-dimensional 'antigens'. Then we use our DTT algorithm [2] for edge detection of time series.

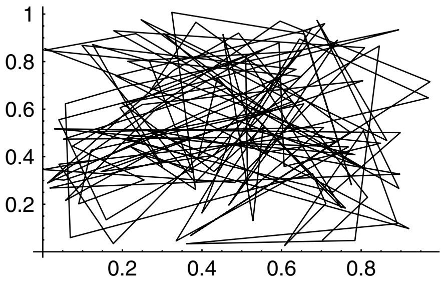

**Figure 1**

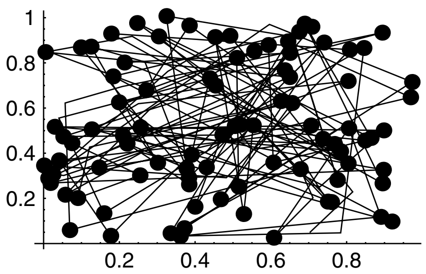

**Figure 2**

Copyright © 2004 John Wiley & Sons, Ltd.

### A method to identify hidden Markov process

A hidden Markov model of a set of data $\{h(1), \ldots, h(N)\}$ is a finite set of probabilities, distribution $B = \{b_1, \ldots, b_{p^{m+1}}\}$, where $p$ is a prime number and $m \geq 1$ is an integer called Markov memory, such that:

1. $\sum_{k=np+1}^{(n+1)p} b_k = 1, \quad$ for any $n = 0, \ldots p^m - 1$,

2. for $M$ satisfying $p^M \geq N > p^{M-1}$,

$$h(j) = \prod_{n=1}^{M} d\left(n, \left[\frac{j}{p^{M-n}}\right] + 1\right), j = 1, \ldots, p^M,$$

where

$$\text{d(n,j)} = \begin{cases} \frac{1}{\text{p}^{\text{n}}}, 1 \leq n \leq m \\ b(Mod(j-1, p^{m+1}) + 1), m < n \leq M \\ j = 1, \ldots p^n \end{cases}$$

and $Mod(m, n)$ gives the remainder of the division of $m$ by $n$.

It is clear that the collection $\{d(n,j), n = 1., M, j = 1, \ldots, p^n\}$ has a tree structure with $M$ generations. Obviously, $h(j)$ represents the overall probability of being at the $j$th branch of the $M$th generation with respect to a certain concatenation of the branches of the tree structure.

Now, given a part of a hidden Markov model of length $N$:

$$H = \{h(1), \ldots, h(N)\}$$

we shall detect $p, m$ and $B = \{b_1, \ldots, b_{p^{m+1}}\}$.

Our algorithm is the following:

(a) Let $P$ be the set of all primes. For each $p_i \in P$ we find $M_i$, such that: $p^{M_i} < N < p^{M_i+1}$.

(b) For any Mi, determine the set:

$$S_{p_i} = \{m_j : 2p_i^{m_j+1} < p_i^{M_i}\} = \left\{m_j : m_j < M_i - 1 - \frac{\log 2}{\log p_i}\right\}.$$

(c) For any triple $(p_i, m_j, M_i)$ as above, we define the walks $a_{n,k}(H)$, where:

$$n = 1, \ldots, M_i \text{ and } k = 1, \ldots, p_i^n.$$

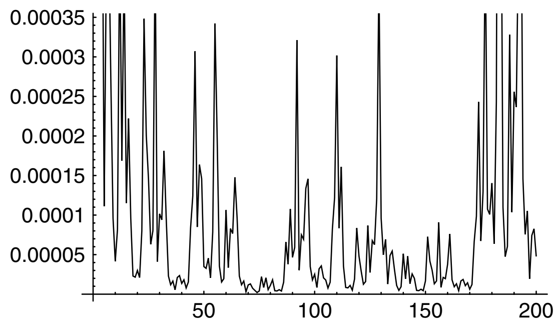

**Figure 3**

(d) For any triple $(p, m, M)$, we compute $b(k) = a_{M,k}(H) - a^{m+1}_{M,k+p}(H)$, where $k = 1, \ldots, p^{M+1}$. If $b(k) = 0$ for any $k$, then select the particular triple $(p,\ m,\ M)$, else we continue with the next triple.

#### Example

Given a part of a Markov data of length 200 (Figure 3), we detect the triple $(p, m, M) = (2, 3, 7)$ and the set of probabilities $B = \{\alpha_{m+1,k}(H), k = 1, \ldots, p^{m+1}\} = \{0.807104$, 0.192896, 0.581991, 0.207806, 0.792194, 0.634231, 0.365769, 0.698161, 0.698161, 0.301839, 0.155554, 0.844446, 0.288346, 0.711654, 0.341626, 0.658374}.

### On measuring the diversity of strings

It is well known that the immune system can effectively recognize a large variety of peptides of viruses. In the case of intrusion of unknown viruses, the immune reaction provides anti-viruses whose peptides differ significantly from what is 'stored' in the 'memory' of the immune system (innate immunity).

In computational analysis a typical measure of diversity is the entropy formula. The entropy of a string written in an alphabet of $r$ letters or digits is given by the formula $\Sigma_i p_i \log(p_i)/\log(r)$, where $p_j$ is the probability of appearance of the letter or digit $j$. This formula is unsatisfactory for measuring the diversity of strings (or collections of strings), because when the digits are almost equidistributed, the entropy is approximately 1. In fact, on a typical RNA we estimated the probabilities: 0.274, 0.192, 0.20 and 0.33 for A, C, G and T, respectively.

Copyright © 2004 John Wiley & Sons, Ltd. 

Using the previous formula we estimate the entropy as 0.983696.

As a consequence, an entropy formula for the diversity of strings should measure the local distribution of digits. Since the usual diversity of strings over an alphabet of $p$ letters depends more on the local information than on the average distribution of digits (because they are usually equidistributed), information or complexity measures based on averaging are not helpful.

In order to estimate the diversity aspects, we consider the distribution of the peptide location in a string. As we will see, the locations of peptides approximately define a fractal-type set. We shall call this set peptide fractal.

We consider a specific peptide written in an alphabet of p letters in a string of length $p^N$. Consider the subset $J$ of the set $\{1, 2, \dots, p^N\}$ indicating the locations of the peptide in a string. We call peptide fractal the sequence $S = \{s(n), n = 1, \dots, p^N\}$, such that $s(n) = 1$ for $n \in J$ and $s(n) = 0$ otherwise.

#### Example

Given the peptide aac and the RNA {aacatgaacaact ...}, its peptide fractal is $\{1, 0, 0, 0, 0, 0, 1, 0, 0, 1, 0, \dots\}$.

Thus, a diversity estimation of strings can be obtained by an approximate Hausdorff dimension of the peptide fractal.

The relation between the Hausdorff dimension (HD) and the Shannon entropy of Markov symbolic shifts is well defined by the Shannon–McMillan–Breiman theorem [3,11]. It identifies the HD of a fractal supporting a singular measure, with the Shannon entropy of a symbolic Markov shift. The Shannon entropy of a symbolic Markov shift with transition matrix $P = \{p_{i,j}\}$ is: $\Sigma_{i,j} p_{i,j} \log(p_{i,j})$.

Applying Bilingsley's formula for HD [3] and using arguments from our previous work [5,6,7,8] for fractals described by non-homogenous Markov processes, we now introduce the entropy formula for peptide fractals S:

$$H_p(S) := \frac{1}{N \log(p^c)} \times \sum_{m=1}^{p^N} \sum_{n=1}^{N} \sum_{j=1}^{p} (a_{n,h(m,k)+j} \log a_{n,h(m,k)+j})$$

where $a_{n,k}$ $(n = 1, \dots N, k = 1, \dots, p^n)$ are the walks of the $p$-adic DTT of $S$, $c$ is the cardinality of S and $h(m,k) := p[m/p^{N-k+1}]$.

We applied this entropy formula to several well-known peptide fractals as the Cantor set and we get the HD with an error less than 3%. On applying this formula to peptide fractals of RNA, we had the following observations.

The peptide fractal entropies of RNA are numbers distributed between 0 and 1 and in a sense characterize the peptide fractal. For large RNA (8000 data), we observed that the entropy on windows (1000 data) is constant with an error less than 3%; in this sense we can say that peptide fractals have self-similarity.

### A new approach for string matching

String matching is a very important subject in the wider domain of text processing. Although data are memorized in various ways, text format remains the main form of information exchange. This is the case, for example, in molecular biology because biological molecules can often be approximated as sequences of nucleotides or amino acids. String matching consists of finding some or all of the occurrences of a string in a text (for more details, see [9]).

In this section we develop a new method for string matching:

- Consider a string $T = \{t_1, t_2, \dots, t_N\}$ written over an alphabet of $m$ letters.
- Associate each different letter of the alphabet to $\{p_1, p_{2,\dots,}p_m\}$, where $p_i$ is the $i$th prime number, thus:

$$\{t_1, t_2, \dots, t_N\} \longrightarrow \{x_1, x_2, \dots, x_N\}$$

  where $x_i$ is one of the first $m$ primes.
- Define the set $Q = \{q_i : q_i = p_i/p_{i+1}, i = 1, 2, \dots N\}$.
- Let $\{t_i, t_{i+1}, \dots, t_{i+j-1}\}$ be a substring of $T$ of length $j$; we define the positive real number:

$$SM(i,j,T) = \prod_{k=i}^{i+j-1} x_k^{q_{k+i-1}} = x_i^{q_1} x_{i+1}^{q_2} \dots x_{i+j-1}^{q_M}, \quad (i = 1, \dots N - j).$$

Copyright © 2004 John Wiley & Sons, Ltd.

Let $W = \{w_1, w_2, \ldots, w_M\}$ and $T = \{t_1, t_2, \ldots, t_N\}$ be two strings written in the same alphabet. Then the equality $SM(i, j, T) = SM(k, j, W)$ $(k = 1, \ldots, M - j)$, implies matching the substrings $\{t_i, t_{i+1}, \ldots, t_{i+j-1}\}$ and $\{w_k, w_{k+1}, \ldots, w_{k+j-1}\}$. This is a consequence of a uniqueness proposition [1].

The algorithm consists of exactly $(N - j + 1)$ $(M - j + 1)$ calculations and no text comparisons. It has been effectively applied to DNA strings and detected precisely the positions of all matching substrings of the same length.

### Acknowledgements

This research was supported by the European Commission under the EU Project IST-2000-26016, 'Immunocomputing'.

### References

1. Atreas N, Karanikas C. A string matching algorithm based on a new discrete transform (draft).
2. Atreas N, Karanikas C, Tarakanov AO. 2003. Signal processing by an immune type tree transform. In *Lecture Notes in Computer Science*, vol 2787. Springer: Berlin; 111–119.
3. Bilingsley P. 1965. *Ergodic Theory and Information*. Wiley: New York.
4. Browder F. 2002. Reflections on the future of mathematics. *Notices Am Math Soc* **49**(6): 658–662.
5. Bisbas A, Karanikas C. 1994. Dimension and entropy of a non-ergodic Markovian process and its relation to Rademacher Riesz products. *Monatshefte Math* **118**: 21–32.
6. Bisbas A, Karanikas C. 1990. On the Hausdorff dimension of Rademacher Riesz products. *Monatshefte Math* **110**: 15–21.
7. Bisbas A, Karanikas C, Moran W. 1997. Tameness for the distribution of sums of Markov random variables. *Math Proc Camb Phil Soc* **1**(1): 115–127.
8. Bisbas A, Karanikas C, Proios G. 1998. On the distribution of digits dyadic expansions. *Results Math* **3–4**: 330–341.
9. Charras C, Lecroq T. Handbook of exact string-matching algorithms. **http://www-igm.univ-mlv.fr/~lecroq/string/**.
10. Karanikas C, Proios G. 2003. A non-linear discrete transform for pattern recognition of discrete chaotic systems. *Chaos Solitions Fractals* **17**: 195–201.
11. Khinchin AI. 1957. *Mathematical Foundations of Information Theory*. Dover: Publications, Inc.: New York.
12. Tarakanov A, Goncharova L, Gupalova T, Kvachev S, Sukhorukov A. 2002. Immunocomputing for bioarrays. Proceedings of the 1st International Conference on Artificial Immune Systems, ICARIS-2002, University of Kent at Canterbury, UK; 32–40.
13. Tarakanov A, Skormin V. 2002. Pattern recognition by immunocomputing. World Congress on Computational Intelligence, CEC-2002, Honolulu, Hawaii, **1**: 938–943.

Copyright © 2004 John Wiley & Sons, Ltd. 

